# Work environment factors in coping with patient death among Spanish nurses: a cross-sectional survey*

**DOI:** 10.1590/1518-8345.3279.3234

**Published:** 2020-04-17

**Authors:** Maria Povedano-Jimenez, Genoveva Granados-Gamez, Maria Paz Garcia-Caro

**Affiliations:** 1 University of Granada, Facultad Ciencias de la Salud, Granada, Andalucia, Spain.; 2 University of Almeria, Ciencias de la Salud, Almeria, Andalucia, Spain.

**Keywords:** Coping Skills, Nursing, End-of-life Care, Practice Environment, Evidence-Based Practice, Occupational Stress, Adaptação, Enfermagem, Cuidados no Final da Vida, Ambiente de Prática, Prática Baseada em Evidências, Estresse Ocupacional, Afrontamiento, Enfermería, Cuidado al Final de la Vida, Entorno Laboral, Práctica Basada en la Evidencia, Estrés Ocupacional

## Abstract

**Objective:**

to explore self-perception competence among Spanish nurses dealing with patient death and its relationship with work environment, evidence-based practice, and occupational stress.

**Method:**

a cross-sectional web-based survey collected information from a convenience sample of 534 nurses from professional Spanish Colleges who answered four validated questionnaires: Coping with Death Scale, Practice Environment Scale of the Nursing Work Index, Perception of Evidence-Based Practice (EBP) and Nursing Stress Scale.

**Results:**

a total of 79% of the participants were women, the average age was 40 years old, 38% had a postgraduate degree and 77% worked in public health settings. Many nurses evaluated their work environment as unfavorable (66%), reported high occupational stress (83.5±14.9), and had high scores on knowledge/skills in EBP (47.9±11.3). However, 61.2% of them perceived an optimal coping (>157 score). The multivariate logistic model indicated positive associations with work environment and EBP characteristics (OR: 1.30, *p*=0.054; OR: 1.04, *p*=0.007; OR: 1.13, *p*<0.001, respectively) but negative associations with occupational stress and short work experience (OR: 0.98, *p=*0.0043; OR: 0.74, *p*<0.002, respectively). These factors explained 23.1% of the coping variance (*p*<0.001).

**Conclusion:**

although most nurses perceived optimal coping, the situation could be enhanced by modifying several contextual factors. The identification of these factors would improve the quality of end-of-life care by facilitating nursing management.

## Abstract (pt)

**Objetivo:**

explorar a capacidade de autopercepção entre enfermeiros espanhóis que lidam com a morte do paciente e sua relação com o ambiente de trabalho, a prática baseada em evidências e o estresse ocupacional.

**Método:**

pesquisa transversal pela internet coletou informações de uma amostra de conveniência de 534 enfermeiros das Faculdades Profissionais Espanholas que responderam a quatro questionários: Escala de Enfrentamento da Morte, Escala Ambiente de Trabalho da Prática de Enfermagem, Percepção da Prática Baseada em Evidências (EBP, acrônimo em inglês) e Escala de Estresse em Enfermagem.

**Resultados:**

79% das participantes eram mulheres com média de 40 anos, 38% tinham pós-graduação e 77% trabalhavam em ambiente de saúde pública. Muitos enfermeiros avaliaram seu ambiente de trabalho como desfavorável (66%), relataram alto estresse ocupacional (83,5 ± 14,9) e altas pontuações em conhecimento/habilidades em EBP (47,9 ± 11,3). No entanto, 61,2% deles consideraram um enfrentamento ótimo (pontuação >157). O modelo logístico multivariado indicou associações positivas com o ambiente de trabalho e as características da EBP (OR: 1,30, *p* = 0,054; OR: 1,04, *p* = 0,007; OR: 1,13, *p* < 0,001, respectivamente), mas negativas com estresse ocupacional e curta experiência de trabalho (OR: 0,98, *p* = 0,0043; OR: 0,74, *p* < 0,002, respectivamente). Esses fatores explicaram 23,1% da variância de enfrentamento (*p* < 0,001).

**Conclusão:**

embora a maioria dos enfermeiros considerasse um enfrentamento ótimo, a situação poderia ser melhorada pela modificação de vários fatores contextuais. A identificação desses fatores melhoraria a qualidade da assistência no final da vida, facilitando a gestão da enfermagem.

## Abstract (es)

**Objetivo:**

explorar el afrontamiento autopercibido por los profesionales españoles de enfermería para trabajar con pacientes moribundos y su relación con el entorno laboral, la práctica clínica y el estrés ocupacional.

**Método:**

estudio observacional transversal vía internet de una muestra de conveniencia con 534 enfermeros reclutados a través de colegios profesionales españoles que respondieron a cuatro cuestionarios validados: Escala de Afrontamiento de la Muerte, Entorno de la Práctica Enfermera, Práctica Basada en la Evidencia en Enfermería (PBE) y Escala de Estrés para Enfermería.

**Resultados:**

la mayoría de los participantes fueron mujeres (79%), de 40 años de media, con estudios de posgrado (38%) y trabajando en el sistema público (77%). Gran parte evaluó su ambiente de trabajo como desfavorable (66%), mostrando estrés ocupacional (83,5 ± 14,9) y altas puntuaciones en conocimiento/habilidades-PBE (47,9 ± 11,3). Sin embargo, el 61,2% autopercibía un afrontamiento óptimo (> 157 puntos). El análisis de regresión logística multivariante mostró que el afrontamiento se asociaba positivamente con el entorno laboral y dos subescalas-PBE (OR: 1,30, p= 0,054; OR: 1,04, p= 0,007; OR: 1,13, p <0,001, respectivamente), pero negativamente con el estrés ocupacional y la poca experiencia laboral (OR: 0,98, p= 0,0043; OR: 0,74, p <0,002, respectivamente). Estos factores explicaban el 23,1% de la varianza (p <0.001).

**Conclusión:**

aunque la mayoría de los enfermeros percibían un afrontamiento óptimo, la situación podría mejorarse modificando algunos factores. La identificación de estos factores mejoraría la calidad de la atención de los pacientes al final de la vida, facilitando la gestión de enfermería.

## Introduction

Regardless of the field of professional care, a dying patient is an emotionally difficult phenomenon^(^ 1 ^)^ that can influence how professionals cope with death. Thus, nurses must feel confident about not only their ability to effectively take care of dying patients^(^ 2 ^)^ but also their ability to maintain their own well-being^(^ 1 ^)^. Skills for coping with patients’ death and favorable work-environment conditions are associated with the quality of end-of-life care. These skills are essential to reduce dissatisfaction and stress^(^ 3 ^)^.

Some authors^(^ 4 ^)^ refer to the complex multimorbidity during end-of-life care, while others^(^ 2 ^)^ state that hospital management does not commit to the necessary strategies to ensure that nurses feel they are able to face this aspect of their work without difficulty. Therefore, to improve end-of-life care, it is important to know the professionals’ perception of coping with death, attitudes about the use of research, characteristics of the work environment^(^ 5 ^)^, and the degree of stress experienced during end-of-life care.

Nurses may express a variety of emotions and attitudes related to end-of-life care^(^ 1 ^,^ 6 ^)^, which are particularly stressful because they are constantly interpreting grief or death as a personal failure^(^ 7 ^)^. Some authors^(^ 8 ^)^ stated that nurses require a wide range of skills to properly deal with death and manage their own fears, beliefs, and attitudes towards dying patients^(^ 9 ^)^.

Inadequate coping with death decreases job satisfaction^(^ 10 ^)^ and increases nurses’ stress, negatively affecting the quality of care^(^ 11 ^)^. Meanwhile, individuals who perceive themselves as optimally coping with death often assess these difficult situations as challenges and actively face occupational stress^(^ 12 ^)^.

Occupational stress in palliative care is identified as the combination of the problems involved in caring for others, the lack of self-care strategies, and organizational factors^(^ 13 ^)^. In addition, health professionals tend to have high occupational stress rates in situations of greater clinical demand, long working hours, and poor manager support^(^ 5 ^)^, while a favorable work-environment is essential to minimize the negative impact of working with dying patients^(^ 14 ^)^.

Previously, specialized nurses in emergency and palliative-care settings were a strong predictor of the perception of coping during end-of-life care^(^ 6 ^)^. Today, special interest is growing in experiential learning because constant exposure to the process of dying helps nurses learn about the care of terminal patients^(^ 15 ^)^. Insufficient nursing knowledge of research^(^ 16 ^)^ was improved by educational projects and evidence-based practice guides for the care of dying patients and their families^(^ 17 ^)^. Barriers such as gaps in the knowledge of research and the feeling of a lack of support and leadership on the part of nurses make it difficult to implement evidence-based care in practice^(^ 18 ^)^.

Specifically, intensive-care professionals recognize the lack of end-of-life education and training as an obstacle to quality care^(^ 19 ^)^. The health institutions^(^ 20 ^)^ and the increased awareness among palliative-care professionals are the most influential forces for overcoming barriers in the use and development of research with regard to clinical nursing and, thus, improving end-of-life care. Evidence is available concerning the influence of the practice environment, occupational stress, and evidence-based practice in end-of-life care. Thus, knowing whether these factors are related to nurses’ perception when coping with dying patients can be central for nursing managers to minimize the emotional impact^(^ 21 ^)^ and promote high-quality end-of-life care. However, no studies were found on whether all nursing staff members’ perceptions of these factors were associated with coping with death. The aim of the present study is to explore self-perception competence among Spanish nurses dealing with patient death and its relationship with work environment, evidence-based practice, and occupational stress.

## Method

A cross-sectional study was designed to include all the professional registered nurses (RN) in any of the Professional Spanish Colleges of Nursing, employed in different institutions (general healthcare settings, ICU, primary health, palliative care, emergency, surgery, psychiatric units, etc.) with different complexities of care. These Professional Colleges are associations of professionals protected by State Law and recognized by public and private institutions that represent and defend the professional interests of the members.

The recruitment was made using nonrandomized convenience sampling through all Professional Spanish Colleges of Nursing after the study was approved by the regional Bioethics Committee and the Research Committee of the Professional College of Nursing. All the registered nurses received a letter of invitation to participate in the study, as well as the instructions to fill out the set of self-administered questionnaires. The inclusion criteria were as follows: to be a nurse currently belonging to a Professional Spanish College of Nursing, to be able to read and write Spanish and to have access to the Internet. Informed consent was required from all the participants. The sample size was based on the total Spanish RN population (274,817) as of 31 December 2014 (Spanish National Institute of Statistics), resulting in a Type I error of 5% (step up of 384 participants). A final sample included 534 nurses who answered the online surveys.

After being given access to the detailed information of the study, nurses gave their written informed consent and participated individually and anonymously. They provided data through a set of questionnaires on Google Drive between February 2014 and April 2015.

All the administrators of the Professional Colleges of Nursing agreed to provide the link to the online survey website. The study followed the principles of the Declaration of Helsinki and was approved by all the ethics committees of Professional College of Nursing.

Participants provided sociodemographic information. Demographic and work-related data included age, sex, educational level (graduate and postgraduate degree), years of nursing practice, work setting [primary and home care, general hospitalization unit (e.g., internal medicine and surgery) and/or specialized (e.g., oncology, gynecology, pediatrics) and critical care and emergency], and type of health center (public, private or co-public). In addition, they completed four specific questionnaires related to their self-perceived professional competence in dealing with death: the Coping with Death Scale, Practice Environment Scale of the Nursing Work Index, Perception of Evidence-Based Practice and Nursing Stress Scale, as follows:

*A. Coping with Death Scale (CDS)* ^(^ 22 ^)^: This scale characterizes the self-perceived level of coping with dying patients. We used the Spanish version^(^ 23 ^)^ that was previously validated among Spanish palliative care professionals^(^ 24 ^)^ and consists of 30 items scored on a 7-point Likert-type scale [from 1 (totally disagree) to 7 (totally agree)], with a score range of 30 to 210. A total score lower than 105 indicates inadequate coping, while a score greater than 157 represents optimal coping^(^ 23 ^)^. A Cronbach’s alpha value of 0.824 was previously reported among Spanish nursing students^(^ 23 ^)^.
*B. Practice Environment Scale of the Nursing Work Index (PES-NWI):* This questionnaire, previously validated in Spanish nurses working in a clinical practice context (with a Cronbach’s alpha of 0.90)^(^ 25 ^)^, was used to characterize the nature of professional nursing practice in the original magnet hospitals. This scale consists of a total of 32 items classified into five subscales: 1) “nurse participation in hospital affairs” (8 items); 2) “nursing foundations for quality of care” (9 items); 3) “nurse manager ability, leadership and support of nurses” (4 items); 4) “staffing and resource adequacy” (4 items); and 5) “interprofessional relations and joint practice” (7 items). All items can be answered on a Likert-type scale [from 1 (strongly disagree) to 4 (strongly agree)]. Each subscale score was calculated as the average of the subscale item responses. Moreover, the total PES-NWI score was calculated as the mean of the five subscale scores. Values above 2.5 on at least four out of the five subscales account for “favorable”, “mixed” is represented by scores ≥ 2.5 on two subscales and “unfavorable” is represented by scores ≥ 2.5 on one subscale or no subscales^(^ 26 ^)^.
*C. Perception of Evidence-Based Practice Questionnaire (EBPQ):* Professional skill at using better knowledge for decision making was addressed by the Spanish version of the Evidence-Based Practice (EBP) questionnaire^(^ 27 ^)^ that consists of 19 items structured into three subscales: 1) “practice of EBP” (6 items); 2) “attitude towards EBP” (3 items) and 3) “knowledge/skills associated with EBP” (10 items)^(^ 28 ^)^. Cronbach’s alpha was 0.87 for Spanish clinical nurses, 0.929 for the practice subscale, 0.722 for the attitude subscale, and 0.916 for the knowledge/skills subscale^(^ 27 ^)^. All items are evaluated from 1 (totally disagree) to 7 (totally agree) score. High scores indicate more positive attitudes and greater use and knowledge of clinical effectiveness and EBP.
*D. Nursing Stress Scale* (NSS): The Nursing Stress Scale questionnaire assesses and detects different potentially stressful situations in nursing^(^ 29 ^)^. The validated Spanish version, with good reliability (Cronbach’s α value of 0.89)^(^ 30 ^)^, was used. This scale consists of 34 items with four possible answers (1 = never to 4 = very frequently). The total scores ranged from 34 (minimum stress level) to 136 (maximum stress level).

A database was automatically organized after each participant completed the questionnaires on Google Drive. Demographic and work-related factors of the sample were analyzed through descriptive statistics. The interquartile range (IQR) was calculated for the mean of the dimensions evaluated by the scales. The associations between quantitative variables were determined with Pearson’s correlation coefficient. Kruskal-Wallis and Mann-Whitney U tests were used to compare variables that did not follow a normal distribution. Simple linear regression was used to analyze the individual predictive value of the evaluated dimensions on the Coping with Death Scale. Finally, multiple linear and logistic regression models were applied using the stepwise procedure to select the best set of predictors of the perception of coping with death. All analyses were performed with a Type I error of 5% and the associated confidence interval (CI) for each parameter. The data analysis was calculated using the statistical package SPSS version 23 (SPSS Inc., Chicago, IL, USA).

## Results

The descriptive sociodemographic characteristics of the study population (n=534 nurses), according to their self-perceived professional competence in dealing with death, are shown in Table 1. The majority of the participants (78.7%) were female, and the average age was 39.7 years (range 22-65 years); 38% had more than 21 years of work experience, 66% had a graduate degree, and most were working in a public health setting (77%), mainly in general hospitals (27.7%) and primary and home-care centers (27.7%) (Table 1). More than half of the responder nurses (61.2%) reported their perception of their coping with death as optimal (>157 score). Compared with older nurses, young nurses more frequently reported inadequate coping (p<0.001). Moreover, being male, having more years of nursing experience, and working in public health clinical settings were also positively and significantly related to self-perceived coping with death (p< 0.05).

**Table 1:** Sociodemographic characteristics of the participating Spanish nurses recruited between February 2014 and April 2015 (n=534)

Variables	n (%)	Coping with Death Scale		p	Inadequate coping ≤105	Optimal coping ≥157	p
		Mean	SE*		n (%)	n (%)	
Age (years)							
≤30	150 (28.1)	128.7	27.6		41 (38.3)	77 (23.5)	
31-42	162 (30.3)	135.8	28.0	<0.001	35 (32.7)	94 (28.7)	<0.001
43-54	151 (28.3)	142.9	29.1		27 (25.2)	110 (33.6)	
55-65	71 (13.3)	142.1	2.9		4 (3.7)	46 (14.1)	
Gender							
Male	114 (21.3)	142.6	28.4	0.006	15 (14.0)	80 (24.5)	0.06
Female	420 (78.7)	135.0	28.4		92 (85.9)	247 (75.5)	
Nursing experience							
< 10 years	214 (40.1)	129.9	27.0	<0.001	56 (52.3)	110 (33.6)	0.003
10-20 years	118 (22.1)	138.2	29.3		22 (20.6)	76 (23.2)	
> 20 years	202 (37.8)	142.9	28.2		29 (27.1)	141 (43.1)	
Academic degree							
Graduate	354 (66.3)	135.1	28.4	0.038	74 (69.2)	207 (63.3)	0.156
Postgraduate	180 (33.7)	139.7	28.8		33 (30.8)	120 (36.7)	
Health-care setting							
Critical care & Emergency	121 (22.7)	135.2	30.8		32 (29.9)	70 (21.4)	
General Hospitalization Unit	117 (21.9)	138.9	27.4	0.883	18 (16.8)	75 (22.9)	0.532
Specialized Hospitalization Unit	148 (27.7)	137.5	28.6		28 (26.2)	91 (27.8)	
Primary care	148 (27.7)	135.2	27.5		29 (27.1)	91 (27.8)	
Health center							
Public	411 (77.0)	138.0	28.8	0.008	77 (72.0)	261 (79.8)	0.026
Private	90 (16.9)	128.9	27.2		26 (24.3)	42 (12.8)	
Co-Public	33 (6.2)	141.4	26.3		4 (3.7)	24 (7.3)	

* SE = Standard Error

Many nurses (66%) evaluated the quality of their work environment as unfavorable (data not shown), and only one out of the five subscales, “interprofessional relations and joint practice”, was perceived as favorable (2.5 ± 0.8; Table 2). Regarding self-rating of the implementation of EBP, the highest score was found in the knowledge/skills subscale (47.9 ± 11.3). In this respect, the best scores were found among nurses with postgraduate training for the three different factors of the EBP (data not shown), compared with those with less education. Moreover, more than half of the participants also reported high stress levels (83.5 ± 14.9) during end-of-life care (Table 2).

**Table 2:** Scale and subscale scores from the Coping with Death, PES-NWI*, EBPQ^†^ and Nursing Stress questionnaires among the participating Spanish nurses recruited between February 2014 and April 2015 (n =534)

Questionnaire variables	M^‡^	SE^§^	IQR^||^	
Coping With Death overall score	136.7	28.6	134.2	139.1
Inadequate coping (n=107)	95.3	10.7	93.3	97.4
Optimal coping (n=327)	155.3	16.8	153.6	157.3
PES-NWI* overall score	2.2	0.5	2.2	2.3
Professional participation in matters of the institution	2.1	0.6	2.0	2.1
Nursing foundation of the quality of care provided	2.4	0.6	2.4	2.5
Leadership and support to nursing professionals by nurse managers	2.2	0.8	2.2	2.3
Size of staff and adequacy of human resources	2.0	0.7	1.9	2.0
Interprofessional relations and joint practice	2.5	0.8	2.5	2.6
EBPQ^†^				
Practice of EBP^¶^	26.6	7.1	26.0	27.2
Attitude towards EBP^¶^	16.8	3.7	16.5	17.2
Knowledge/Skills associated with EBP^¶^	47.9	11.3	46.9	48.8
Nursing Stress Scale	83.5	14.9	82.2	84.7

* PES-NWI = Practice Environment Scale of the Nursing Work Index;

† EBPQ = Questionnaire on the Perception of Evidence-Based Practice;

‡ M = Mean;

§ SE = Standard Error;

|| IQR = Interquartile Range;

¶ EBP = Evidence-based Practice

Pearson’s correlation showed that age (r= 0.182, p<0.001), the total score for nursing-practice environment (r= 0.171, p <0.001), and “interprofessional relations and joint practice” subscale (r= 0.172, p<0.001) were significantly and positively correlated with coping with death. We also found significant positive correlations with the scores of the three subscales of EBPQ, in favor of nurses with optimal self-perceived professional competence in dealing with dying patients (practice r=0.262; attitude r=0.387; knowledge/skills r=0.240; p<0.001). However, occupational nursing stressful situations and coping with death showed a significant and negative relationship (r= -0.240, p<0.001) as did occupational stress and the work environment (r= -0.341, p<0.001). Positive and significant relationships were also found between the characteristics of the work environment and EBP, highlighting “nursing foundations for quality of care” with practice (r= 161, p<0.001) and attitude towards EBP (r= 0.114, p<0.01) (data not shown).

The multivariate comparison of the PES-NWI factors indicated a significant effect of coping with death (*F*10; 1056 = 2.481; *p* = 0.006; η^2^ = 0.023). Participants with perceptions of inadequate ability to cope with death scored lower on “staffing and resource adequacy” (1.7 ± 0.6) than participants with neutral or optimal self-perceived professional competence in dealing with death (2.0 ± 0.7). The multivariate comparison of the EBP factors also showed a significant effect on self-perceived professional competence in dealing with death (*F*6; 1060 = 13.317; *p* <0.001; η^2^ = 0.070). Those participants with optimal perceived ability to cope with death scored higher on all EBP factors and reported less stress (81.1 ± 15.1) than did participants with inadequate or neutral perceived ability to cope with dying patients (Table 3).

**Table 3:** Associations between coping with death and PES-NWI*, EBPQ^†^ and Nursing Stress questionnaires, among the participating Spanish nurses, recruited between February 2014 and April 2015 (n=534)

Variables	Inadequate coping (<33) N = 107		Optimal coping (>66) N = 327		F Test^§^		
	Mean	SE^‡^	Mean	SE^‡^	F2;531^§^	p^||^	η^2¶^
Age	36.3	10.5	40.9	11.3	6.529	0.002	0.024
PES-NWI* overall score	2.1	0.5	2.3	0.5	9.433	<0.001	0.034
Professional participation in matters of the institution	1.9	0.6	2.1	0.6	4.950	0.007	0.018
Nursing foundation of the quality of care provided	2.3	0.5	2.5	0.6	5.534	0.004	0.020
Leadership and support to nursing professionals by nurse managers	2.0	0.7	2.3	0.8	6.148	0.002	0.023
Size of staff and adequacy of human resources	1.7	0.6	2.0	0.7	6.309	0.002	0.023
Interprofessional relations and joint practice	2.3	0.7	2.6	0.8	8.215	<0.001	0.030
EBPQ Practice of EBP**	24.4	6.9	27.7	6.9	10.290	<0.001	0.037
Attitude towards EBP**	14.5	4.6	17.8	2.8	38.960	<0.001	0.128
Knowledge/Skills associated with EBP**	44.4	12.3	49.7	10.7	11.446	<0.001	0.041
Nursing Stress Scale	88.4	12.3	81.1	15.1	11.826	<0.001	0.043

* PES-NWI = Practice Environment Scale of the Nursing Work Index;

† EBPQ = Questionnaire on the Perception of Evidence-Based Practice;

‡ SE = Standard Error;

§ F Test = Value Distribution Test;

|| p-value;

¶ η^2^ = Eta-square;

** EBP = Evidence-based Practice

The multivariate regression models, after the application of the variable selection method stepwise predictors, showed that some socio-demographic and work environment characteristics, as well as EBP and occupational stress, were related to coping with death, although only explaining 23.1% of the coping variance (*p*< 0.001) (Table 4). The multivariate linear regression model indicated positive associations with work environment and EBP characteristics (β= 0.12, *p*< 0.05; β= 0.16, *p*= 0.001; β= 0.29, *p*= 0.001, respectively) but negative associations with occupational stress (β= -0.10, *p*< 0.015). A positive attitude towards EBP was the factor with the greatest predictive capacity (β = 0.29, *p*= 0.001), indicating that a higher score on this factor predicts a higher score on coping with death. In addition to nursing occupational stress, short working experience also showed a negative slope, with scoring lower with regard to perceived coping with death associated with less than 10 years of nursing practice (β = -0.18, p <0.001). associated with less than 10 years of nursing practice (β = -0.18, *p* <0.001).

**Table 4:** Multivariate lineal regression model of Coping with Death Scale (Spanish nurses' study during 2014-2015)

Variables	Β*	p^†^	95%CI^‡^	
Constant			70.61	111.95
Interprofessional relations and joint practice	0.12	0.004	1.30	6.98
Practice of EBP^§^	0.16	0.001	0.32	0.96
Attitude towards EBP^§^	0.29	0.001	1.63	2.86
Nursing Stress	-0.10	0.015	-0.35	-0.04
Sex (male)	0.07	0.052	-0.03	10.46
Prof Exp (<10 years)	-0.18	0.001	-15.72	-5.68
Prof Exp (10-20 years)	-0.05	0.277	-8.92	2.56

* Β = Beta;

† p value;

‡ CI = Confidence Interval;

§ EBP = Evidence-based Practice

In the same way, when the outcome was dichotomized, the multivariate regression logistic model also showed that some socio-demographic and work environment characteristics, as well as EBP and occupational stress, were related to coping with death (Table 5). Thus, the multivariate model indicated positive associations with work environment (interprofessional relations) and EBP characteristics (practice and attitude) but negative associations with nursing occupational stress. Years of experience and training were also determinant factors of optimally coping with death rather than age. In the logistic model, being a male was not a determining factor of self-perceived professional competence in dealing with death.

**Table 5:** Multivariate regression logistic model of Coping with Death Scale (Spanish nurses' study during 2014-2015)

Variables	OR*	p^†^	95%CI^‡^	
Constant			0.075	4.981
Interprofessional relations and joint practice	1.30	0.054	0.995	1.698
Practice of EBP^§^	1.04	0.007	1.011	1.072
Attitude towards EBP^§^	1.13	0.000	1.068	1.189
Nursing Stress	0.98	0.043	0.971	0.999
Sex (male)	1.47	0.148	0.873	2.463
Prof Exp (<10 years)	0.74	0.002	0.295	0.760
Prof Exp (10-20 years)	0.61	0.295	0.428	1.294

* OR = Odds Ratio;

† p value;

‡ CI = Confidence Interval;

§ EBP = Evidence-based Practice

## Discussion

In our study population, the findings relate several sociodemographic and occupational characteristics to the nurses’ self-perceived professional competence in dealing with death. Specifically, age (older than 31 years of age), gender (being male), education (postgraduate education training) and work experience (having more than 10 yrs. nursing experience) increased the coping with death score. In addition, the results of the questionnaires implied that four different variables were also important for assessing self-perceived professional competence in dealing with death: 1) collegial nurse-doctor relations related to work environment; 2) the individual’s attitude towards EBP, which includes perceived barriers such as workload and personal judgments; 3) the practice related to the implementation of EBP and to individual patient care; and 4) occupational stress.

Different previous studies have corroborated these findings^(^ 31 ^-^ 36 ^)^. According to these studies, males are rational, decisive, and resilient when working in challenging situations, such as caring for dying patients^(^ 31 ^)^; moreover, males often have their own approach to coping with difficult emotions and maintaining their own well-being because male stereotypes of self-sufficiency and competitiveness influence their practices and experiences^(^ 32 ^)^.

Some studies also emphasize the benefit of having longer nursing experience, fuller training in end-of-life care^(^ 33 ^)^, and exposure to situations of suffering such as dying^(^ 34 ^)^. This provides knowledge and skills for improved coping with dying patients in the future, potentially reaching the point of feeling comfortable^(^ 6 ^)^. Nurses with less than 10 years of experience were more vulnerable and exhausted, had higher levels of depersonalization, and had lower levels of personal fulfillment^(^ 35 ^)^. However, at the same time, nurses with more years of experience were able to keep a distance and set boundaries in end-of-life care^(^ 36 ^)^. These findings also fit with previous evidence pertaining to “interprofessional relations and joint practice”^(^ 34 ^)^.

In relation to the perception among Spanish nurses concerning their work environment, two-thirds of the study population considered the work environment unfavorable because their evaluations were lower than the magnet hospital scores. However, the EBP was assessed according to the nurses’ knowledge more than their practice, indicating a discouraging work context. Regarding factors least valued within the PES-NWI scale, a personnel shortage is a restrictive element to fulfilling professional roles and responsibilities^(^ 37 ^)^. A supportive work environment among colleagues is helpful in dealing with end-of-life care^(^ 38 ^)^. This perceived unfavorable work environment could also be related to the economic crisis that Spanish health professionals are lately experiencing^(^ 39 ^)^. Additionally, the high workload in dying patient care demands the same conditions of care as before the start of the crisis and has not adapted to the shortage of resources^(^ 40 ^)^. A shortage of staff could force a choice between providing comprehensive care to all dying patients or focusing only on the most urgent cases^(^ 41 ^)^, causing frustration and emotional exhaustion among professionals^(^ 42 ^)^.

On the other hand, the low scores on the PES-NWI subscale “nurse participation in hospital affairs” deserve special attention by healthcare administrators because this professional participation in matters of organizational performance positively influences efficiency and effectiveness at the professional team level^(^ 43 ^)^, and it was observed that more than half of the professionals reported high levels of participation in hospital affairs in the situations posed.

The positive association found between the attitude towards and practice of EBP and the perceived ability to cope with death suggests the importance of having well-founded knowledge of professional safety, that is, to perceive oneself as having the ability to face the death of others. Several studies have indicated that additional training programs help to adequately address dying patients^(^ 44 ^)^. It should be noted that some institutions have managed to bolster the confidence of nurses to provide the best care to oncological patients after going through EBP educational programs^(^ 45 ^)^. However, there are still barriers to the offer of ongoing education for nurses participating in end-of-life care^(^ 10 ^)^.

The negative association between occupational stress and ability to cope with death, regardless of the nursing workplace, appeared in previous findings^(^ 38 ^,^ 46 ^)^, indicating that compassion fatigue limits coping with the final moments of a patient’s life.

Finally, our findings corroborate the relationship between the dimensions of the professional context analyzed and the ability to cope with death. Specifically, it was found that the worst scores in all dimensions of the context analyzed were related to the lowest level of the ability to cope with death; however, not all of the dimensions analyzed had the same predictive value for self-perceived competence dealing with death. The model of predictors showed that the following factors influenced the nurses’ ability to cope with dying patients, with moderate explanatory power: sex (male), nursing experience (less than 10 years and between 10 and 20 years), “collegial nurse-doctor relations”, the perception of the work-environment, practice factors, attitude towards EBP and nursing stress. Several studies have noted that the positive association found between interprofessional relations and joint practice and the nurse’s self-perceived professional competence in dealing with death was the main source of emotional support for improving nurse care for dying patients^(^ 46 ^-^ 48 ^)^.

This study has several shortcomings and strengths. It has an observational design, so we could not definitively establish causal relationships. Although the study population could be considered representative of the Spanish population (a country and a culture), the recruitment system limited any generalization. Most of the participants were female, which is in line with the gender distribution in the Spanish nursing profession. All the questionnaires used were validated but may have led to a bias in information gathering due to a lack of knowledge or other unidentified factors. Additionally, the questionnaires were completed online, restricting the web survey to the nurses who had internet access. Nevertheless, these questionnaires have been used in numerous studies among the Spanish population to reduce information bias. The sample size was large, the study subjects were from different regions of Spain, and many distinct factors were considered simultaneously. We should also highlight that to minimize selection bias, the study was performed not only in palliative care or oncology but also in other nursing specialties.

According to our results, this type of survey should be used to detect which nurses may perceive high levels of self-competence in end-of-life care. It is important that nursing professionals become comfortable with dying patients in their work, which implies that attitudes, social awareness, or personal opinions may also influence coping strategies. Moreover, healthcare institutions must be involved to identify the factors that influence the quality of life of the health professional and, therefore, the quality of their practice. Nursing education should also promote specific training to reinforce coping strategies at the end of life or the management of feelings towards the patient who will die to avoid negative influences on the work of future professionals.

## Conclusion

Despite the challenges that a nurse faces in an environment with dying patients, this situation has not been a determinant for the optimal level of perceived ability to cope with death. The determining factors are age, years of experience and training. In a predominantly female environment, being male proves to be a determining factor of an optimal ability to cope with death and one of the factors that predicts the self-perceived professional competence in dealing with death. Future studies should thoroughly analyze these gender differences. All the elements of the professional context analyzed were found to influence the level of the nurses’ ability to cope with death; however, the moderate explanatory power suggests the need to consider other elements not considered in this study. Finally, given the importance of the professional team, the nursing administration plays a key role in providing better practice environments, by facilitating the use and training of research in the workplace and identifying occupational stress.
